# A randomised clinical trial on a comprehensive geriatric assessment and intensive home follow-up after hospital discharge: the Transitional Care Bridge

**DOI:** 10.1186/1472-6963-10-296

**Published:** 2010-10-29

**Authors:** Bianca M Buurman, Juliette L Parlevliet, Bob AJ van Deelen, Rob J de Haan, Sophia E de Rooij

**Affiliations:** 1 Department of Internal Medicine and Geriatrics, Academic Medical Center, Room F4-108, P.O. Box 22660, 1100 DD Amsterdam, The Netherlands; 2 Department of Internal Medicine, Flevo Hospital, Hospitaalweg 1, 1315 RA Almere, The Netherlands; 3 Clinical Research Unit, Academic Medical Center, P.O. Box 22660, 1100 DD Amsterdam, The Netherlands

## Abstract

**Background:**

Older patients are at high risk for poor outcomes after acute hospital admission. The mortality rate in these patients is approximately 20%, whereas 30% of the survivors decline in their level of activities of daily living (ADL) functioning three months after hospital discharge. Most diseases and geriatric conditions that contribute to poor outcomes could be subject to pro-active intervention; not only during hospitalization, but also after discharge. This paper presents the design of a randomised controlled clinical trial concerning the effect of a pro-active, multi-component, nurse-led transitional care program following patients for six months after hospital admission.

**Methods/Design:**

Three hospitals in the Netherlands will participate in the multi-centre, double-blind, randomised clinical trial comparing a pro-active multi-component nurse-led transitional care program to usual care after discharge. All patients acutely admitted to the Department of Internal Medicine who are 65 years and older, hospitalised for at least 48 hours and are at risk for functional decline are invited to participate in the study. All patients will receive integrated geriatric care by a geriatric consultation team during hospital admission. Randomization, which will be stratified by study site and cognitive impairment, will be conducted during admission. The intervention group will receive the transitional care bridge program, consisting of a handover moment with a community care Care Nurse (CN) during hospital admission and five home visits after discharge. The control group will receive 'care as usual' after discharge. The main outcome is the level of ADL functioning six months after discharge compared to premorbid functioning measured with the Katz ADL index. Secondary outcomes include; survival, cognitive functioning, quality of life, and health care utilization, satisfaction of the patient and primary care giver with the transitional care bridge program. All outcomes will be measured at three, six and twelve months after discharge. Approximately 674 patients will be enrolled to either the intervention or control group.

**Discussion:**

The study will provide new knowledge on a combined intervention of integrated care during hospital admission, a proactive handover moment before discharge and intensive home visits after discharge.

**Trial registration:**

Trial registration number: NTR 2384

## Background

Hospitalisation is a hazardous event for patients of 65 years and older. Many older people are acutely admitted to the hospital for reasons like an infection or gastrointestinal bleeding. This acute disease is often accompanied by other chronic diseases as well as other impaired health conditions such as delirium, falls and malnutrition which complicate treatment during and after hospital admission [1-4]. The complexity of diseases and other health conditions make older patients prone for adverse hospital outcomes including mortality, institutionalization and functional decline [5,6]. Improving patient safety and prevention of adverse hospital outcomes are considered priorities in these patients.

Functional decline is defined as a deterioration of one or more activities of daily living (ADL) after discharge compared to premorbid ADL functioning, and has become an increasingly important focus of care during and after hospital admission as it is experienced by 15-50% of acutely hospitalized patients [7-9]. Decline in ADL function frequently precedes acute hospital admission [10] and once ADL function is lost, it is difficult to recover [11].

Several approaches to prevent functional decline have been studied. The effect of comprehensive geriatric assessment (CGA), an intervention consisting of screening on the risk for adverse outcomes, a diagnostic assessment on the presence of geriatric conditions and tailor-made interventions provided by a multidisciplinary team has most often been studied, showing mixed results. Studies conducted on specialised geriatric units have demonstrated the effectiveness of the CGA approach [12]. However, in studies on inpatient geriatric consultation services where a multidisciplinary team visits patients on different units, effects differ [13]. Main components of successful studies were targeting interventions to patients at risk for adverse outcomes and following patients after discharge.

Other approaches often studied are 1) intensive discharge planning and home follow-up after discharge [14,15] and 2) transitional care [16]. These approaches demonstrated to be effective to prevent rehospitalisation and length of hospital stay. Most of these studies did not focus on functional outcomes. Studies combining CGA and intensive follow up after discharge are still scarce.

All patients that are included in the present study will receive CGA during their hospital stay. The aim of the present study is to investigate whether a transitional care bridge program following discharge leads to a preservation of physical functioning. The current paper describes the methods that will be used in conducting the study.

## Methods

### Design and setting

Three hospitals in the Netherlands will participate in this multicentre, double-blind, randomised clinical trial (RCT): the Academic Medical Center in Amsterdam(AMC), a 1024-bed university teaching hospital, the Onze Lieve Vrouwe Gasthuis in Amsterdam (OLVG), a 555-bed teaching hospital and the Flevo Hospital in Almere, a 386-bed regional teaching hospital. The transition from hospital to home and home follow-up will be provided by registered nurses affiliated with three home care organisations connected to the hospitals; Cordaan Home Care, Buurtzorg Nederland and Zorggroep Almere. The study is scheduled to start June 1, 2010 and will end after the last patient has been followed up for six months. We expect the study to end May 31, 2013.

### Participants

All patients of 65 years and over acutely admitted to the department of internal medicine of the three participating hospitals and hospitalised for at least 48 hours are invited to participate. These patients are screened for the risk for functional decline using the Identification of Seniors at Risk-Hospitalized Patient (ISAR-HP, table 1, in review). Patients with a score of two or more on this screening instrument are at high risk for functional decline and eligible for inclusion.

**Table 1:** Scorecard: Identification of Seniors At Risk - Hospitalized Patients (ISAR-HP)

ISAR-HP		
	YES	NO
1. Before hospital admission, did you need assistance for IADL (e.g., assistance in housekeeping, preparing meals, shopping, etc.) on a regular basis?	1	0
2. Do you use a walking device (e.g., a cane, walking frame, crutches, etc.)?	2	0
3. Do you need assistance for traveling?	1	0
4. Did you pursue education after age 14?	0	1
Total score (circled figures)		

Total score 0 or 1 = not at risk

Total score ≥ 2 = patient is at risk for functional decline

Patients are excluded if they are 1) terminally ill, 2) do not give informed consent 3) transferred to Intensive Care, Coronary Care Unit or to another ward within 48 hours after hospital admission, 4) came from another department or another hospital 5) not fluent in the Dutch languages or 5) came from a nursing home. Patients presenting with cognitive impairment may participate in the study.

### Approvals

The study was approved by the AMC's Medical Ethics Committee which forms part of the University of Amsterdam in the Netherlands (protocol ID MEC10/082). Participants will provide written informed consent prior to enrolment. In case of cognitive impairment written informed consent will be obtained by the patients' primary care giver. Recruitment procedures will be conducted in accordance with the Dutch Medical Research Involving Human Subjects Act and the WMA Declaration of Helsinki.

### Randomisation and blinding

**Figure 1:**
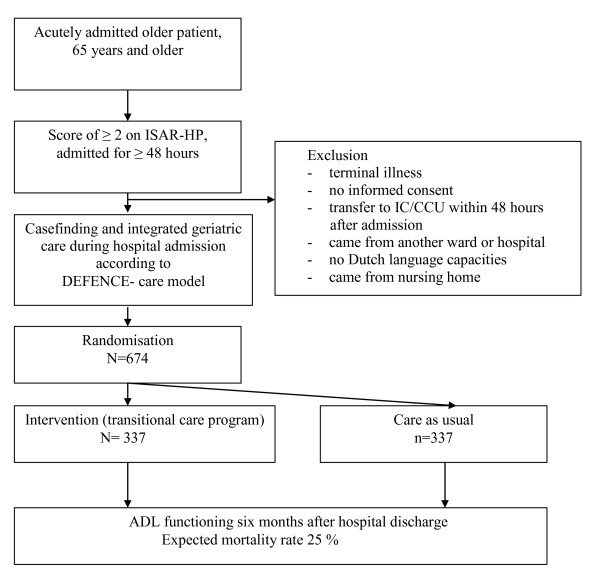
**Flow chart of patient selection and randomisation**.

After obtaining informed consent and baseline assessments, patients will be randomised into the intervention or control group (figure 1). The randomisation procedure will be website-based, using permuted blocks and stratified by study centre and level of cognitive functioning (Mini-Mental State examination of ≥ 24 versus MMSE scores of < 24).

The study will be double-blinded as patients will be blinded to the intervention by using a postponed informed consent procedure described by Boter *et al *[17]. This informed consent procedure is chosen because we expect to introduce bias by informing all patients about the intervention of study. Patients in the control group could be unsatisfied with not being allocated to the intervention group, whereas patients in the intervention group could give better ratings to the intervention. For example, patients might score higher out of loyalty to the community care nurse that helped them. Patients in the intervention group are further informed about the care coordination after discharge but not about that this is the actual intervention to be studied. The control group is not informed about the intervention. After termination of the study, patients in both study groups will receive written information concerning the complete research question by means of a letter.

A research nurse blinded to the intervention will conduct all follow up assessments. The multidisciplinary teams in the hospitals and the community care nurses are not blinded to randomization.

### Hospital care provided to all patients included in the study

The geriatric consultation team in each of the hospitals will consist of a geriatrician, Clinical Nurse Specialist (CNS) in Geriatrics, Registered Nurse (RN), physiotherapist and a dietician. The RN will visit the participating wards on a daily basis (except for the weekends) to screen patients for eligibility. Patients at high risk for functional decline, as determined by the ISAR-HP, will receive a systematic comprehensive geriatric assessment initially performed by the RN (table 2). The assessment will start with screening on delirium, malnutrition, ADL functions, mobility and fall risk. In cognitive impaired patients, part of the CGA will be conducted by interviewing the primary care giver. The primary care giver will always be interviewed about burden of care givers and the amount of time spent helping the patient at home before admission.

**Table 2:** Content of the Comprehensive Geriatric Assessment (CGA) performed at hospital admission

Domain	Question or instrument in CGA	Condition/ Disease
**SOMATIC**		
1. Mobility and stability	Have you been fallen once or more in the past six months?	Falls
	Do you experience dizziness?	Dizziness
	Have you ever had a fracture?	Osteoporosis risk
		
2. Medication	Only if patients use medication Do you experience difficulties or side effect with medication use?	Medication safety and side effects
	Polypharmacy defined as the use or five or more different medications	Polypharmacy
	Medication adherence with the questionnaire of Aburuz [24]	Medication adherence
		
3. Nutrition	Short Nutritional Assessment Questionnaire (SNAQ) [25]	Malnutrition
	Was the patient dehydrated at admission?	Dehydration
	Difficulties with swallowing?	Swallowing disturbance
		Obesity or underweight
	Body mass index	Oral hygiene
	Do you have pain in your mouth?	
		
4. Urine and fecal problems	Do you experience urine incontinence? Do you experience fecal incontinence	Incontinence
	Do you experience obstipation?	Obstipation
	Do you have an indwelling urinary catheter? Did you already have this at home?	Indwelling urinary catheter use
		
5. Skin	Do you have pressure ulcer(s)?	Pressure ulcer
		
6. Pain	Visual analogue scale for pain [26]	Pain
		
7. Allergy	Are you allergic?	Allergy
		
PSYCHOLOGICAL		
1. Delirium	Have you ever experienced a delirium?	Delirium
	Confusement Assessment Method [27]	
		
2. Depression	Geriatric depression Scale [28,29]	Depression
		
3. Cognition	Mini-Mental State Examination [30]	Cognitive impairement
		
4. Anxiety	Do you feel anxious?	Anxiety
		
5. Dependency	Do you smoke?	Alcohol, smoking and medication use
	Do you use alcohol	
	Do you use benzodiazepines?	
		
FUNCTIONAL		
1. ADL functioning	Katz ADL index score [19]	ADL dependency
		
		
2. IADL functioning	IADL questions of Lawton and Brody [31]	IADL dependency
		
3. mobility difficulty	Are you using a walking aid?	Mobility difficulty
		
4. Hearing	Do you experience difficulties with hearing, despite the use of a hearing aid?	Hearing impairment
		
5. Visual	Do you experience difficulties with your vision, despite the use of glasses?	Visual impairment
		
6. Sleep	Do you experience problems with sleeping?	Sleeping disorder
	Do you use sleeping medication? If yes, how often?	
		
SOCIAL		
1. Loneliness	De Jong Gierveld-questionnaire [32]	Loneliness
		
2. Burden of care giver	Care giver extension of the Minimal Data set	Burden of care giver
		
3. Health related quality of life	EQ-6 D [22]	Health related quality of life

The questions or instruments are a starting point for further diagnostics or treatment; if necessary a more intensive screening will be conducted by the multidisciplinary team

Empowerment of patients and primary care givers is an important topic in this study. After the CGA, patients or their primary caregiver will be asked to indicate which problems should be given highest priority for treatment. Furthermore, attention will be given to patients' most important goals to be achieved during and after hospital admission. This information will be taken into account when discussing the outcome of the CGA with the geriatrician and CNS.

A team meeting with the geriatric consultation team will result in a tailor-made care- and treatment plan which will be discussed with the patient and primary care giver. If patients did not give priority to a certain problem and the geriatric consultation team considers the problem relevant to treat, the patient and primary care giver will be informed about why the team advices to have a certain condition treated and what are the treatment options. Thus, the patient and primary care giver can make a well-informed decision about the care and treatment plan.

The care and treatment plan will be carried out during admission in accordance with the medical and nursing care at the ward where the patient is admitted. If necessary, other disciplines will be consulted, such as a pharmacist or occupational therapist.

### The intervention

#### The transitional care bridge program

The overall transitional care bridge program consists of two steps; 1] the discharge procedure concerning the transition of care and 2] the continuation of the integrated care in the primary care by a community care nurse.

##### Step 1: The experimental discharge procedure including transition of care

This step concerns the transfer of care from hospital to primary care. The care during this phase and the second phase will be provided by a community care nurse (CN). The CN is a bachelor level educated nurse with a special focus on the elderly. The CN can work in a general practice, within a home care organisation or can be affiliated to a nursing home.

The transition from hospital to home consists of the following sub-steps.

(a) A handover for the care and treatment plan is made by the geriatric consultancy team and is coordinated by the CNS as part of the integrated care plan at least two days before discharge from hospital. This plan includes the ongoing interventions and recommendations for care in the primary care setting.

(b) The transition of care-plan made by the CNS will be offered to the primary care CN of the patient who is visiting the patient in hospital before discharge.

(c) After visiting patient in the hospital, the CN will discuss the care plan with the (substitute) General Practitioner (GP) of the patient.

(e) Guided by the care and treatment plan handed over from the hospital and depending on the needs of the patients and caregiver, additional support will be enabled by the CN (for example consisting of dietician, occupational therapist, the elderly welfare consultant, physiotherapist and/or pharmacist).

It is expected that approximately 6% of the patients leaving hospital are not discharged home but will be admitted in an intermediate care facility or rehabilitation care in a nursing home. In this subgroup a CN from the nursing home or rehabilitation centre will visit the patient in the hospital.

##### Step 2: Experimental continuation of care in primary care

The intervention consists of the following steps and will mainly be provided by the CN after discharge.

(a) The CN visits the patient within two days after hospital discharge at home. In this first visit, special attention is paid to medication and appropriateness of care arranged during hospital admission.

(b) The second visit is two weeks after hospital discharge where the CN (re)assesses the care- and treatment plan and where needed the CN makes adaptations to the plan and discusses clarity of the medication regimen from the hospital. In this visit, social functioning, participation and existing care needs will be discussed with the patient.

(c) The CN will ensure continuation at home of the interventions started in the hospital. When necessary, the CN also coordinates indications for new interventions.

(d) The CN maintain contacts with other practitioners (e.g. occupational therapy, dieticians, pharmacists, physiotherapy, elderly welfare consultant etc.) in consultation with the general physician.

(e) The CN identifies new care/treatment needs (e.g. imminent (re) admission to hospital) in consultation with the GP

(f) The CN as transition coach also promotes the empowerment of patients and carers by including the provision of psycho-education on the identified geriatric conditions and providing ancillary services such as leisure, day treatment and care [18].

For patients discharged to a nursing home or rehabilitation centre, the same steps will be conducted but the CN visit the patients in these settings and contacts the Nursing Home Physician (NHP) for consultation

After 2, 6, 12 and 24 weeks, the CN visits the patients and evaluates the care- and treatment plan, the impact and the (intended) results. The results are discussed in regular meetings of the primary care geriatric consultancy team. This team consist of the GP (or NHP) and the CN, and depending on patients care needs it is complemented with a consultant pharmacist, a primary care physiotherapist, occupational therapist, elderly welfare consultant, dietician and/or a social worker. An in-hospital consultant (geriatrician) is appointed at hospital discharge that can also easily be consulted by the CN, GP or NHP.

The GP or the NHP remains the final responsible director for the medical care of the patient.

### Control group

Patients allocated to the control group will receive 'care as usual' after discharge. This consists of a discharge home after admission. The medical resident of the hospital will send a discharge letter to the GP of the patient that most often is received two weeks after discharge. Additional care can be arranged with a home care organisation and consists of help in conducting ADL. Most patients are followed up six weeks after discharge at the outpatient department. The consult mainly consists of laboratory testing and focuses on the disease(s) patients were discharged with.

### Evidence based care and uniformity of care provided

The currently applied interventions in the integrated care plan are all evidence based or based on current best practice in the hospital and in the community. For the purpose of the present study an evidence based toolkit has been constructed which describes the present state-of-the-art in care and treatment of the geriatric conditions. All geriatric conditions in this toolkit are worked out in the same structure: goal to achieve with a certain condition, the theoretical background (prevalence, risk factors), screening in the hospital and community care (which question or validated instrument can be applied), action plan, further diagnostics and how to apply these, evidence based interventions (including when to consult other disciplines) and financing care.

The toolkit will be used to create uniformity in screening, diagnostics and interventions and is the basic for the tailor-made care plan (available at http://www.defencestudy.nl) [in Dutch].

### Efforts to decrease the burden for very ill patients and cognitive impaired patients

Attrition of frail older persons is a problem frequently met in trials conducted in this patient population [7]. In this randomised clinical trial, we have made efforts to decrease all possible burden for these frail patients in order to make it possible to include this group and to minimize drop-outs.

At admission, the inclusion procedure for very ill patients and cognitive impaired patients is limited. This short assessment consists of screening on five geriatric conditions: delirium, malnutrition, activities of daily living functioning, mobility and fall risk. This assessment is chosen because these geriatric conditions contribute most to adverse outcomes can be easily observed or screened and are most prone to early intervention. If patients are not able to answer question, the primary care giver will be interviewed.

To build a strong and trusting relationship between the CN and the patient and family, the starting point of the intervention will be during hospital admission by visiting patients during hospital admission. That way the CN is a person more familiar to the patient and primary care giver and they both know that the CN is informed about the care provided in the hospital.

After discharge, all patients in the intervention group will be visited in their own home to minimize the burden of the visits.

### Outcomes

#### Primary outcome

The primary outcome measure is the level of ADL functioning six months after discharge from the hospital compared to premorbid functioning two weeks prior to hospital admission. The level of ADL functioning will be measured with the Katz ADL index score [19]. The Katz ADL index score consists of 6 items, with score range from 0 to 6, with a higher score indicating more impairment in ADL. At both time points, the questionnaire will be filled in by the same person (patient or proxy, depending on cognitive impairment)

#### Secondary outcomes

Secondary outcomes will be measured at baseline, three months, six months and one year after discharge from hospital by a research nurse who was blinded to the nature of the transitional care program. Secondary outcomes include:

(1) Mortality

(2) ADL functioning, as measured with the ALDS, a validated, Item Response Theory-based generic and validated continuous scale with a score range between 0 and 100, with a lower score expressing more impairment in daily functioning [20]

(3) Cognitive functioning and health-related quality of life (IQCODE-SF [21] and EQ-6 D [22])

(4) Experiences with providing care by primary care givers and burden of primary care givers (with the primary care giver extension of the minimal dataset)

(5) Satisfaction of patients and primary care givers with the care provided

(6) Health care utilization (economic extension of the Minimal Dataset with care issues such as institutionalization, rehospitalisation and/or visits to the emergency department of the hospital, amount of care provided by professional care and primary care giver)

### Process evaluation

In addition to the primary and secondary outcomes additional (semi-) qualitative data will be collected that will give an insight in the feasibility of the transitional care bridge intervention at the professional and AMC geriatric network level. Qualitative data will be analyzed in relation to primary care and hospital derived factors that the future implementation of the care (might) impede or promote.

### Sample size calculation

In determining the appropriate group size in order to demonstrate a significant intervention effect on the primary endpoint, we used Cohen's effect size *d *to determine the difference between the patients' KATZ ADL index scores on the before and after measurement and divided by the SD of the difference scores of the control group as a benchmark for assessing the relative magnitude of ALDS score differences between both strategies. Although an effect size of 0.25 can be defined as small, such a difference in Katz ADL scores may be clinically important.

We have demonstrated that with a total of 506 patients (253 patients per treatment arm) we are able to statistically detect (power 80%, two-sided alpha of 5%) a minimal effect size on the Katz ADL index score. To allow for attrition due to mortality, which is expected to be 25% six months after admission, a total of 674 patients will be included in the trial.

### Data analysis

Statistical analyses will be based on an intention-to-treat principle. Baseline assessments and outcome parameters will be summarized using simple descriptive statistics. The main analysis focuses on a comparison between the trial intervention and control group of the primary outcome, the Katz ADL index score. The same approach will be used with regard to the secondary outcome parameters, including survival rates. Survival data will be additionally analyzed using Kaplan-Meier survival curves and the log-rank test.

We will perform a predefined subgroup analysis for discharge destination (patients discharged to home versus nursing home). In all analyses statistical uncertainties will be quantified via corresponding 95% confidence intervals. Separate subgroup analysis will also be conducted on patients at intermediate (ISAR-HP score of two or three) and high risk for functional decline (ISAR-HP score of four or five). Finally, process outcome data will be analyzed qualitatively within the theoretical framework of the adaptive implementation model [23].

## Discussion

With an ageing population in many countries and increasing life expectancy, there is an urgent need to improve outcomes of hospital admission. Preservation of decline in ADL functions and preventing institutionalization have become a more important focus of care, rather than only minimizing mortality rates. Several approaches to improve hospital outcomes have been studied focusing on comprehensive geriatric assessment and intensive home follow up after discharge. The present RCT combines these approaches to provide optimal care during hospital admission and to improve ADL functioning after discharge.

The study is conducted as part of the National Care for the Elderly program in which special emphasis is given to regional geriatric care networks. The current study will provide information on the feasibility of the intervention, collaboration between hospitals and primary care as well as on structural funding of care.

## Abbreviations

(ADL): activities of daily living; (ALDS): AMC linear disability scale; (ISAR-HP): Identification of Seniors at Risk-Hospitalized Patient; (CGA): comprehensive geriatric assessment; (CN): community care nurse; (CNS): clinical nurse specialist in geriatrics; (DEFENCE): Develop strategies Enabling Frail Elders New Complications to Evade; (EQ-6D) Six-Dimensional EuroQol instrument; (ES): effect size; (GEM): Geriatric Evaluation and Management units; (GP): general practitioner; (IADL): Instrumental Activities of Daily living; (IQCODE): informant questionnaire on cognitive decline in the elderly; (MMSE): Minimal Mental State Examination; (RCT): randomised clinical trial; (RN): registered nurse; (NHP): nursing home physician; (SD): standard deviation.

## Competing interests

The authors declare that they have no competing interests.

## Authors' contributions

BB drafted the manuscript and wrote the protocol for the Medical Ethics Committee. SR drafted the research proposal. JP, BD, RH, SR critically reviewed the manuscript and protocol for the Medical Ethics Committee. BB, JP, BD and RH reviewed the research proposal that was sent to the funding organization. RH was involved in the methodological construct of the study. All authors read and approved the final version of the manuscript

## Funding

This study is funded by a grant from by the Netherlands Organization for Health Research and Development (Zon MW), National Care for the Elderly program, grant number 311020201

## Pre-publication history

The pre-publication history for this paper can be accessed here:

http://www.biomedcentral.com/1472-6963/10/296/prepub
